# Editorial: Laparoscopic surgery in colorectal cancer

**DOI:** 10.3389/fonc.2022.960730

**Published:** 2022-09-01

**Authors:** Wenqing Jia, Xi Cheng, Ren Zhao

**Affiliations:** ^1^ Department of General Surgery, Ruijin Hospital, Shanghai Jiao Tong University School of Medicine, Shanghai, China; ^2^ Shanghai Institute of Digestive surgery, Ruijin Hospital, Shanghai Jiao Tong University School of Medicine, Shanghai, China

**Keywords:** single incision laparoscopic surgery, robot-assisted surgery, colorectal cancer, laparoscopic surgery, editorial

Laparoscopic surgery for colorectal cancer has evolved quickly and enthusiastically applied in clinical works for almost two decades (1). Regular laparoscopic colorectal surgery usually need four or five incisions, in recent years, efforts have been spent to further minimize the trauma, reduce postoperative pain, and improve cosmetic effect (2). Innovations like natural orifice transluminal endoscopic surgery (NOTES), transanal total mesorectal excision (taTME), robotic surgery, and single incision laparoscopic surgery (SILS) has been developed to reach the goal of “scarless” surgery, which represents the state-of-the-art phenomenon in the field (3, 4). Additionally, burgeoning instruments, single-incision port devices, and robot platforms open up a new scenario of CRC surgery (5, 6).

Under this circumstance, this Research Topic collected 9 scientific studies focused on surgical methods and experience of minimally invasive surgery, especially single incision laparoscopic surgery (SILS) and robotic surgery.

A growing knowledge has revealed that port subtraction benefits CRC patients in multiple aspects without a sacrifice of operative and oncology safety. Zhang et al. proved that laparoscopic right hemicolectomy (LRC) conducted through three ports harvested more lymph nodes and caused less blood loss compared with LRC performed with five ports in a retrospective clinical trial. Port-reduced surgery demonstrated a latent advantage in long-term outcomes such as overall survival (OS), which required more convincing evidence. According to a well-designed randomized controlled trial conducted by Song et al. in 193 CRC patients, SILS performed by experienced surgeons reduced postoperative pain and depicted good short-term outcomes as well as cosmetic effects compared with conventional laparoscopic surgery. The long-term outcomes and complications of SILS such as incisional hernia are expected to be revealed in sequential research with a low loss of follow-up rate and high quality of evaluation.

The transanal approach has also been developed with the aim of minimizing vulnus. Despite mini-residual risks, transanal endoscopic microsurgery (TEM) is a choice for early-stage rectal cancer. Tang et al. have launched the first prospective multicenter randomized trial to compare the risk of local recurrence and total survival of TEM followed by radiotherapy and TME in T2N0M0 distal rectal cancer patients. Guo et al. summarized the evolution and narrated the latest research status of taTME, a surgery that provided an accurate exposure of the mesorectal plane and direct vision of the distal resection margin. They also analyzed the disputations about taTME and provided a dialectical view on its future.

Regardless of multiple approaches to CRC surgery, dissection of No.253 lymph nodes to a certain extent and preservation of autonomic nerve is a shared issue, with all schools of thought contending for attention. Zheng et al. proposed a novel technique for nerve-sparing high ligation of the inferior mesenteric artery (IMA) called intrasheath separation of the IMA and partial preservation of the left IMA sheath along with the left trunk of the inferior mesenteric plexus (IMP), based on anatomical evidence of the spatial relationship between IMA and IMP. While Li et al. believe that the IMP nerve plane can be separated from IMA by enforcing traction and anti-traction, also it is identified as the dorsal border of station 253 nodes, thus creating a “nerve plane orientation” technique. Both of them have heuristic value in avoidance of postoperative urogenital dysfunction caused by latent autonomic nerve damage in surgery.

Wang et al. took an in-depth look at the quality of surgical resection in laparoscopic, robotic, and transanal total mesorectal excision for mid-/low rectal cancer, by means of a Bayesian network meta-analysis. This systemic review prompt researchers to deepen their insight into the pros and cons of various minimally invasive surgical approaches and facilitate clinical decision-making.

Postoperative medical care and timely intervention of other adjuvant therapies are also significant for patient recovery. Wang et al. disclosed the significant role of enhanced recovery after surgery (ERAS) as an “icing on the cake” combined with SILS, leading to earlier dietary resumption and shorter hospital stays of CRC patients after SILS. Kumara et al. discovered a phenomenon in which keratinocyte growth factor (KGF), an FGF family protein mainly produced by mesenchymal cells, is elevated significantly for a 5-week course after minimally invasive colorectal resection probably as a consequence of acute inflammatory response and wound-healing. However, its latent role in tumor recurrence and metastasis raises the question of whether perioperative anticancer treatments are essential and feasible, and this deserves further exploration.

In a nutshell, the papers included in this Research Topic describe the brand-new development of laparoscopic surgery in colorectal cancer. We’d like to express our sincere gratitude to all authors, editors, and reviewers of all these publications, as well as the editorial team at Frontiers for their devotion and assistance in the process of reviewing and publishing this Research Topic. The future trend points to a combination of SILS and robotic surgical platform, and the next decade promises to illuminate more in-depth aspects of “scarless” surgery in colorectal cancer treatment.

## Author contributions

All authors listed have made a substantial, direct, and intellectual contribution to the work and approved it for publication.

## Conflict of interest

The authors declare that the research was conducted in the absence of any commercial or financial relationships that could be construed as a potential conflict of interest.

## Publisher’s note

All claims expressed in this article are solely those of the authors and do not necessarily represent those of their affiliated organizations, or those of the publisher, the editors and the reviewers. Any product that may be evaluated in this article, or claim that may be made by its manufacturer, is not guaranteed or endorsed by the publisher.
